# Phishing alert! A Cas9-based method reveals the identity of promoter-bound transcription factors

**DOI:** 10.1093/plphys/kiad020

**Published:** 2023-01-18

**Authors:** Marieke Dubois

**Affiliations:** Department of Plant Biotechnology and Bioinformatics, Ghent University, 9000 Ghent, Belgium; VIB Center for Plant Systems Biology, 9000 Ghent, Belgium

Transcriptional regulation is a key process for controlling gene expression spatially and temporally. Transcription factors (TFs) are the central proteins that bind to promoters to activate or repress gene expression. TFs often fine-tune gene expression by interacting with other proteins on a promoter, resulting in, for example, tissue-specific expression, re-enforced activation, or timely response to environmental signals. Gene Ontology analysis has identified no fewer than ∼1,700 DNA-binding TFs in Arabidopsis (*Arabidopsis thaliana*), ∼2,800 in rapeseed (*Brassica napus*), ∼2,400 in strawberry (*Fragaria × ananassa*), and ∼1,700 in soybean (*Glycine max*) and tobacco (*Nicotiana tabacum*) (Van Bel et al., 2022). Unraveling TF-DNA interactions is crucial for better understanding how TFs act (together) to regulate the expression of their target genes, which are often not yet identified.

During the last decades, two complementary strategies have been developed to uncover interactions between TFs and DNA. The first strategy centers on the TFs and aims to identify the DNA sequences bound to a TF of interest. The most widely used method in this strategy is Chromatin Immuno Precipitation (ChIP), in which the TF of interest and the DNA associated with it are cross-linked chemically *in planta*. Subsequently, the TF is purified through affinity purification using antibodies against the TF or an epitope. Upon decrosslinking, the TF-bound DNA fragments are identified by PCR or sequencing. The second strategy uses DNA as a starting point to identify the proteins bound to a specific part of the DNA, often the promoter of the gene of interest. An example of such a method is yeast-one-hybrid (Y1H), in which a promoter of interest is fused to a reporter gene and screened against a library of TFs expressed in yeast cells. A caveat of Y1H is that it cannot identify the TFs bound to the DNA in the native context. In vivo alternatives have also been described previously, including Proteomics of Isolated CHromatin (PiCh, Déjardin and Kingston, 2009) and reverse-ChIP (Wen et al., 2020). More recently, Genomic Locus Proteomics (GLoPro) has been developed in mammalian cells, combining CRISPR-based targeting of a DNA sequence with proximity labeling around the targeted site (Myers et al., 2018). Although these DNA-centered methods are appealing and innovative, to date, their efficiencies have been too low to effectively identify in vivo promoter-bound TFs in plants.

In the current issue of *Plant Physiology*, Wang et al. (2022) present a DNA-centered method to capture the TFs bound to a promoter of interest in plants (Figure 1). The “Reverse-ChIP based on CRISPR-dCas9” (R-ChIP-dCas9) method is based on the ability of CRISPR/Cas9 to target specific DNA sequences in a guide-RNA-dependent manner. Cas9 was engineered to maintain its DNA binding capacity without its endonuclease activity (dead Cas9, dCas9). The dCas9 is fused a Strep-tag, enabling affinity purification of DNA-protein complexes. A single vector carrying the gRNA that targets a promoter of interest and the dCas9-Strep-coding sequence can be transiently transformed into any plant species transformable by *Agrobacterium tumefaciens*. Subsequently, all proteins and DNA are crosslinked by a formaldehyde treatment and the DNA is fragmented to ∼1 kb. Strep-tag purification enables isolating the dCas9, along with the targeted DNA sequence and all TFs bound to the target DNA. Upon decrosslinking, the eluted proteins are identified by mass spectrometry.

**Figure 1:**
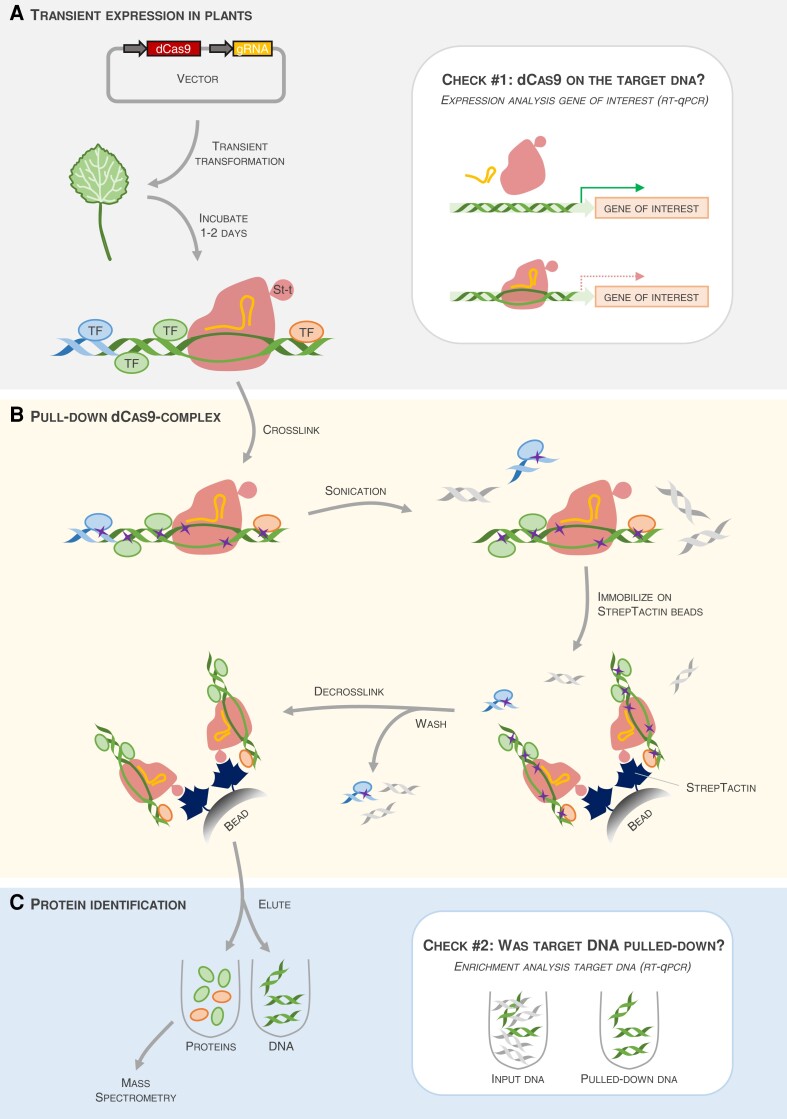
Pipeline of the reverse ChIP based on CRISPR-dCas9 (R-ChIP-dCas9) method. A, The method presented by Wang et al. (2022) is based on transient expression of nuclease-dead Cas9 (dCas9) tagged with a Strep-tag II (St-t) and a guide-RNA (gRNA) complementary to the target DNA of interest. One to two days after transient transformation in plants, the dCas9 (red) binds to the DNA region of interest (here, a promoter, the green double-stranded helix) via the gRNA (orange curved line). This step can be verified (Check #1) by reverse transcription quantitative PCR (RT-qPCR), as the gene of interest driven by this promoter should have lower expression if dCas9 has properly bound to the target region. B, dCas9, DNA, and all proteins bound to the DNA (here, only TFs are shown) are crosslinked (purple crosses) by chemical treatment. DNA is fragmented into pieces of ∼1 kb. Subsequently, the Strep-tag-carrying dCas9 is pulled-down using StrepTactin beads, and the unbound proteins (blue ellipses) and DNA fragments (blue or gray double-stranded helices) are washed away. Finally, the dCas9-complexes are decrosslinked. C, To identify the TFs bound to the DNA region of interest, proteins and DNA are eluted. Via RT-qPCR, enrichment of the targeted DNA in the eluted sample, as compared to the abundance in the input sample, can be verified (Check #2). If the pull-down of the targeted DNA was successful, the eluted proteins can further be identified by mass spectrometry.

The (reverse-)ChIP protocols are often long and tedious; therefore, Wang and colleagues proposed two ways to ensure the success of the different steps (Figure 1). First, measuring the expression level of the gene driven by the targeted promoter serves as a proxy for proper interaction of the dCas9/gRNA complex (Figure 1, Check #1). Promoters targeted by dCas9/gRNA are often repressed, so the expression of the corresponding gene should be lower compared to the control plants that do not contain the gRNA (Wang et al., 2022). The second verification step takes place after dCas9 immunoprecipitation and decrosslinking: the targeted DNA should be strongly enriched in the eluted fraction compared to input fraction containing all fragmented DNA (Figure 1, Check #2).

In the present study, Wang et al. (2022) provided proof-of-concept for R-ChIP-dCas9 in the tree species Asian white birch (*Betula platyphylla*). *BpNAC090* is strongly induced during drought stress, and elevated *BpNAC090* expression in transgenic birch confers drought tolerance, making *BpNAC090*an interesting gene to further study molecularly (Jia et al., 2022). Wang and colleagues used R-ChIP-dCas9 to identify the candidate TFs acting upstream of *BpNAC090*. R-ChIP-dCas9 on the *BpNAC090* promoter yielded 1,741 proteins, of which 390 were nuclear-localized and 32 were annotated TFs. Out of these TFs, five were selected for the validation of the TF-DNA interaction by independent methods including ChIP, Y1H, and electrophoretic mobility shift assays (EMSAs). Overall, all tested TF-DNA interactions identified by R-ChIP-dCas9 on the *BpNAC090* promoter were validated.

The innovative reverse-ChIP method developed by Wang et al. (2022) opens new possibilities for the identification of upstream regulators of a gene of interest. Given the flexibility of the CRISPR system, R-ChIP-dCas9 is applicable to the majority of promoter sequences of all plant species that are transformable by *Agrobacterium tumefaciens* (Wang et al., 2022). Whereas several methods have been successfully developed in the past few decades to detect protein–protein interactions, protein–RNA interactions, and more recently protein–metabolite interactions, there has still been a need for efficient methods to detect DNA–protein interactions *in planta* using DNA as bait. The R-ChIP-dCas9 method, developed and validated in plants, will undoubtedly trigger plant scientists to “phish” for upstream regulators of their favorite genes.

## References

[kiad020-B1] Déjardin J , Kingston RE (2009) Purification of proteins associated with specific genomic loci. Cell 136(1): 175–186 19135898 10.1016/j.cell.2008.11.045 PMC3395431

[kiad020-B2] Jia Y , Niu Y, Zhao H, Wang Z, Gao C, Wang C, Chen S, Wang Y (2022) Hierarchical transcription factor and regulatory network for drought response in Betula platyphylla. Hortic Res 9: uhac040 10.1093/hr/uhac040 PMC9070641 35184174

[kiad020-B3] Myers SA , Wright J, Peckner R, Kalish BT, Zhang F, Carr SA (2018) Discovery of proteins associated with a predefined genomic locus via dCas9-APEX-mediated proximity labeling. Nat Methods 15(6): 437–439 29735997 10.1038/s41592-018-0007-1 PMC6202184

[kiad020-B4] Van Bel M , Silvestri F, Weitz EM, Kreft L, Botzki A, Coppens F, Vandepoele K (2022) PLAZA 5.0: extending the scope and power of comparative and functional genomics in plants. Nucleic Acids Res 50(D1): D1468–D1474 34747486 10.1093/nar/gkab1024 PMC8728282

[kiad020-B5] Wang Z , He Z, Liu Z, Qu M, Gao C, Wang C, Wang Y (2023) A reverse chromatin immunoprecipitation technique based on the CRISPR-dCas9 system. Plant Physiol 191(3): 1505–1519 10.1093/plphys/kiac506 PMC10022611 36305686

[kiad020-B6] Wen X , Wang J, Zhang D, Ding Y, Ji X, Tan Z, Wang Y (2020) Reverse Chromatin Immunoprecipitation (R-ChIP) enables investigation of the upstream regulators of plant genes. Commun Biol 3(1): 770 33318632 10.1038/s42003-020-01500-4 PMC7736860

